# 43 genes support the lungfish-coelacanth grouping related to the closest living relative of tetrapods with the Bayesian method under the coalescence model

**DOI:** 10.1186/1756-0500-4-49

**Published:** 2011-03-07

**Authors:** Yunfeng Shan, Robin Gras

**Affiliations:** 1 School of Computer Science, University of Windsor, 401 Sunset Avenue, Windsor, ON N9B 3P4, Canada; 2 Department of Biological Sciences, University of Windsor, 401 Sunset Avenue, Windsor, ON N9B 3P4, Canada

## Abstract

**Background:**

Since the discovery of the "living fossil" in 1938, the coelacanth (*Latimeria chalumnae) *has generally been considered to be the closest living relative of the land vertebrates, and this is still the prevailing opinion in most general biology textbooks. However, the origin of tetrapods has not been resolved for decades. Three principal hypotheses (lungfish-tetrapod, coelacanth-tetrapod, or lungfish-coelacanth sister group) have been proposed.

**Findings:**

We used the Bayesian method under the coalescence model with the latest published program (Bayesian Estimation of Species Trees, or BEST) to perform a phylogenetic analysis for seven relevant taxa and 43 nuclear protein-coding genes with the jackknife method for taxon sub-sampling. The lungfish-coelacanth sister group was consistently reconstructed with the Bayesian method under the coalescence model in 17 out of 21 taxon sets with a Bayesian posterior probability as high as 99%. Lungfish-tetrapod was only inferred from BCLS and BACLS. Neither coelacanth-tetrapod nor lungfish-coelacanth-tetrapod was recovered out of all 21 taxon sets.

**Conclusions:**

Our results provide strong evidence in favor of accepting the hypothesis that lungfishes and coelacanths form a monophyletic sister-group that is the closest living relative of tetrapods. This clade was supported by high Bayesian posterior probabilities of the branch (a lungfish-coelacanth clade) and high taxon jackknife supports.

## Background

**Figure 1:**
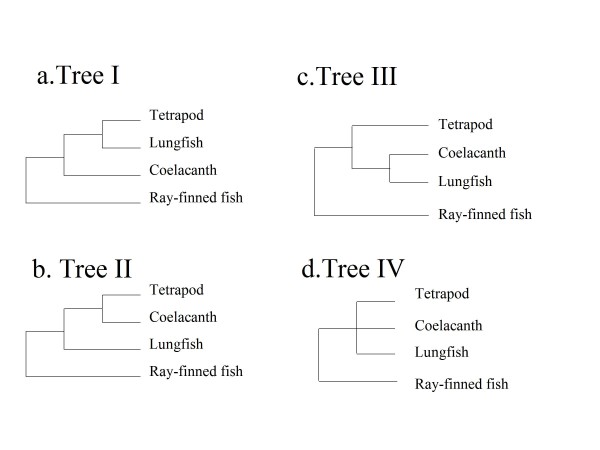
**Four Alternative Phylogenetic Trees among Tetrapod, Coelacanth and Lungfish Lineages**.

The origin of land vertebrates (tetrapods) has not been fully resolved. Since the discovery of the "living fossil" in 1938, *Latimeria chalumnae *[1,2], the last discovered surviving species of a lineage of lobe-finned fish, has generally been considered the closest living relative of the land vertebrates, the missing link between aquatic and terrestrial vertebrates. This is still the prevailing opinion in most general biology textbooks [3]. The origin of tetrapods always has considerable popular interest in public and academic fields since the legendary fish discovery. Three hypotheses have been proposed for the phylogenetic relationship: e.g., lungfish-tetrapod (Hypothesis 1, Figure 1a), coelacanth-tetrapod (Hypothesis 2, Figure 1b), or, lungfish-coelacanth sister group (Hypothesis 3, Figure 1c). The coelacanth-lungfish-tetrapod trichotomy (Figure 1d) is not generally considered a hypothesis.

Based on comparative morphological and paleontological studies, the lungfish were historically thought to be the closest living relatives of tetrapods [4,5], but the coelacanths were purported to have that claim [1,6,7] since its discovery in 1938, whereas the coelacanths and lungfish sister group (Tree III) was also proposed [8-10].

Over the last two decades, single genes and whole mitochondrial genomes have been sequenced with a view to inferring phylogenetic relationships. Lungfish as the closest relatives of tetrapods were supported by single genes [11-15] and mitochondrial whole genomes [16-19], the coelacanth as the closest living sister group of tetrapods was preferred by single genes [20], and coelacanth-lungfish sister group relationship was suggested by the single gene [13] and the mitochondrial whole genome [17,19], while an unresolved coelacanth-lungfish-tetrapod trichotomy was shown by the 12 S rRNA gene [12].

Recently, this question was reinvestigated. The result using 44 genes with the concatenation genome-scale approach [21] was an unresolved trichotomy. Another from two recombination activating genes (Rag1 and Rag2) supported lungfish and not the coelacanth as the closest living relative of the tetrapods [15]. Our previous study provided strong evidence in favor of rejecting Hypothesis 2, but weak evidence to support Hypothesis 3 based on 43 genes with three common phylogenetic methods and three genome-scale approaches [22,23].

Although many morphological, paleontological and molecular phylogenetic studies have attempted to resolve this question, the results have so far not discovered unequivocal evidence as to whether either the coelacanth or the lungfish is the closest living relatives of tetrapods or that both lineages are equally closely related to tetrapods. Therefore, the origin of tetrapods continues to be debated and still is one of the longest standing major questions in vertebrate evolution.

BEST implements a Bayesian hierarchical model to jointly estimate gene trees and the species tree from multilocus sequences [24]. The procedure applies the same substitution models as those used in traditional phylogenetics and coalescent theory to explain genealogical signals from species trees to gene trees and from gene trees to sequence data, forming a complete stochastic model to estimate gene trees, species trees and species divergence times simultaneously [25]. The model is based on the assumption that gene trees are correlated due to being come from a single species tree and therefore should be estimated jointly [25]. It provides a new approach for estimating species phylogenies within the popular Bayesian phylogenetic program MrBayes [26]. BEST was applied to a prominent yeast phylogenomics data set and have shown that it is more efficient in estimating the species tree than concatenation is in estimating the gene tree [27]. With simulation data, analysis of the same DNA sequences by concatenation using either Bayesian or maximum-likelihood methods reconstructed the wrong tree with high confidence, whereas the BEST analysis converged on the correct tree with high confidence [27].

To provide more evidence to resolve the origin of tetrapods, we used here the Bayesian method under the coalescence model with a newly published program (Bayesian Estimation of Species Trees, or BEST) for genome-scale phylogenetic analysis [24-27] and the jackknife method for taxon sub-sampling to analyze all 43 nuclear protein-encoding genes that are currently available in Genbank, having considered the results of our previous study [23] using three other genome-scale approaches with all three commonly used phylogenetic methods together. Seven taxa include Mammal (M), Bird (B), Amphibian (A), Coelacanth (C), Lungfish (L), Ray-finned Fish (R), and Shark (S).

## Materials and methods

### Sequence Collection

The sequences of 43 nuclear protein-encoding genes were downloaded from GenBank through the National Center for Biotechnology Information http://www.ncbi.nlm.nih.gov/ using the program SeqMiner.pl [28]. These 43 genes were previously analyzed using the genome-scale approach of concatenated genes; however, the sequence length of some genes was different [Additional file 1: Supplemental Table S1]. One gene (FSCN1) is omitted because some taxa lack its sequences in GenBank. In order to compare the results with the genome-scale concatenated multiple gene approach [21], the same seven taxa were included: Mammal, Bird, Amphibian, Coelacanth, Lungfish, Ray-finned Fish, and Shark. The species examined included human (*Homo sapiens*), bird (*Gallus gallus*), amphibian (*Xenopus laevis*), coelacanth (mostly *Latimeria chalumnae*, with a few *L. menadoensis*), lungfish (mostly *Protopterus dolloi and P. aethiopicus*) with a few *Neoceratodus forsteri *and *Lepidosiren paradoxa*), ray-finned fishes (*Danio rerio*), and cartilaginous fishes (represented by *Scyliorhinus canicula*) [21].

### Phylogenetic Analysis

Sequences of an individual gene were aligned using ClustalX with default settings [29]. All alignments of single genes were manually edited to exclude insertions or deletions and uncertain positions for further analysis. The BEST phylogenetic analysis software (version 1.1) with the Bayesian method under the coalescence [24] was used for tree inference under the GTR + Γ+ I model and four simultaneous Markov chains for 20 million generations, starting with random initial trees and sampling every 2000 generations. The burnin value was set to 100. The majority rule consensus tree was generated using the remaining trees with posterior probability plotted on each node.

### Taxon Jackknife Sub-sampling

We used a jackknife approach to sub-sample six, five and four taxa from seven taxa with permutation and combination. The debate over taxon sampling has not terminated. On the one hand, the accuracy was enhanced dramatically with the addition of taxa [30]. On the other hand, adding taxa can reduce accuracy and increase the probability of distorting the tree topology [30]. Adding characters can always increase the accuracy [30-32]. So, as many genes as possible should be included. The sequence data of 43 genes that are all currently available in GenBank were used in this study. Sequence data sets are available upon request.

### Chi-square Test

The **s**tatistically significant difference in the Bayesian posterior probabilities for the branch of the lungfish-coelacanth or the taxon jackknife support averages between the six-, five- and four-taxon sets was analyzed by means of the chi-square test.

## Results

**Figure 2:**
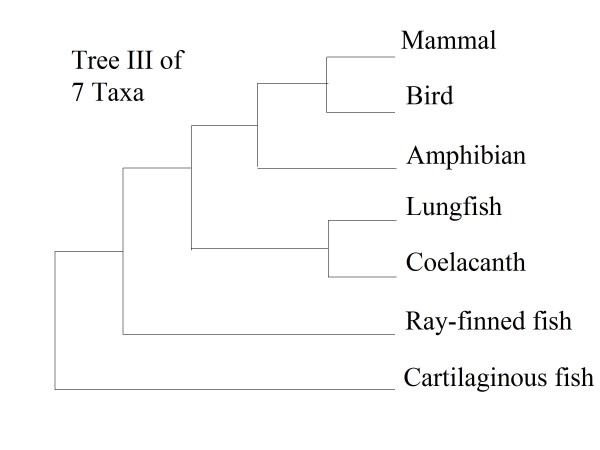
**The Phylogenetic Relationship (Tree III) of 7 Taxa**. Numbers indicated above branches correspond to Bayesian Posterior Probabilities.

Tree III was inferred with 90% Bayesian posterior probability of the branch of lungfish-coelacanth for seven taxon set (Figure 2 and Table 1). Four of five six-taxon sets recovered tree III with Bayesian posterior probabilities for the branch of lungfish-coelacanth ranging from 77 to 93%. The exception was MBCLRS, which recovered an alternative tree (Table 1). Seven of the nine five-taxon sets inferred tree III, but BACLS recovered tree I and ACLRS reconstructed an alternative tree. Although BCLS recovered tree I, all the other five four-taxon sets inferred tree III (Table 1).

**Table 1:** Tree Types, Bayesian Posterior Probability of the Branch of Lungfish-Coelacanth (tree III) or Lungfish-Tetrapods (Tree I) for 7, 6, 5 and 4-Taxon Sets, and Taxon Jackknife Supports with the Bayesian Method under the Coalescence Model

Taxon Set	Tree Type	Probability
7 taxon set		
MBACLRS	III	90%
		
6 taxon sets		
BACLRS	III	90%
MACLRS	III	80%
MBACLR	III	93%
MBACLS	III	77%
MBCLRS	AT	n/a
		
5 taxon sets		
ACLRS	AT	n/a
BACLR	III	49%
BACLS	I	43%
BCLRS	III	49%
MACLR	III	97%
MACLS	III	64%
MBCLR	III	45%
MBCLS	III	82%
MCLRS	III	62%
		
4 taxon sets		
ACLR	III	99%
ACLS	III	67%
BCLR	III	40%
BCLS	I	89%
MCLR	III	73%
MCLS	III	73%

JKF:	III (17/21)	81.00%
	I (2/21)	9.50%
	AT (2/21)	9.50%
	II	0
	IV	0

Notes:

The taxa included: Mammal (M), Bird (B), Amphibian (A), Coelacanth (C), Lungfish (L), Ray-finned Fish (R), and Shark (S); JKF = Taxon jackknife supports (%); AT = alternative tree; n/a = not available.

Taxon jackknife support was 81.0% for tree III, 9.5% for tree I or an alternative tree other than tree I - IV, and zero for tree II and tree IV with the Bayesian method under the coalescence model for all 21 taxon sets (Table 1).

Bayesian posterior probabilities for the branch of lungfish-coelacanth were 85.0, 64.0, and 70.4% and taxon jackknife support averages were 83.3, 77.8, and 80.0% for the six-, five-, and four-taxon sets, respectively (Table 1). The chi-square test showed no significant differences among the taxon sampling sets. The results showed that taxon sampling had no significant effect on phylogenetic inference for the taxon sets.

## Discussion

Tree III is consistently reconstructed with the Bayesian method under the coalescence model in 17 out of 21 taxon sets with a Bayesian posterior probability as high as 99%. Tree I was inferred only from BCLS and BACLS, and two alternative trees were recovered from ACLRS and MBCLRS (Table 1). Therefore, we provide strong evidence to support Hypothesis 3, namely that coelacanths and lungfish form a monophyletic group that is the phylogenetically closest living relatives of tetrapods (Tree III). Our results agree with those of other studies in terms of the morphological, palaeontological and molecular analyses below. The coelacanth and lungfish sister group relationship was supported by the single gene [13] and the whole mitochondrial genome [19], and by the nuclear 28 S ribosomal RNA gene [17]. This relationship was also proposed in comparative morphological and paleontological studies [8-10].

Recently, an investigation using 44 genes with a concatenation genome-scale approach showed an unresolved trichotomy [21]. Another result from two genes supported lungfish and not the coelacanth as the closest living relative of the tetrapods [15]. In our previous study [22,23], tree II received significantly lower support than tree I or tree III and, evidently, lower taxon jackknife probabilities with all the phylogenetic methods and genome-scale approaches. The supports for tree III were significantly higher than those for tree I for only two out of 63 events, and taxon jackknife probabilities for tree III were slightly higher than those for tree I with MP, but the differences in supports and taxon jackknife probabilities between tree III and tree I are not as obvious as those between tree II and tree III/I. Therefore, the results in our previous study provide strong evidence to reject Hypothesis 2 that coelacanth is the closest living relative of tetrapods, but only weak support for Hypothesis 3 based on phylogenetic analysis of 43 genes with those three common methods and those three genome-scale approaches yet at that time [22,23]. Our results in this study also provide further strong evidence in favor of rejecting Hypothesis 2 because none of 21 taxon sets recovers tree II. Recently major palaeontological studies proposed that lungfishes are the closest living relatives of the tetrapods or alternatively, that coelacanths and lungfishes form a monophyletic sister group that is equally closely related to the tetrapods [33,34]. The cause of this puzzle is the fact that the divergence of coelacanth and lungfish happened over a relatively short period within a small (20-30 millions years) window in time around 400 million years ago [3,5]. This results in little time and opportunity for lineage-specific molecular changes to happen, yet considerable time and opportunity for multiple and parallel changes and their accumulation since the origin of these two lineages [3]. For this challenging phylogenetic question, therefore, it was very difficult to achieve high resolution using ad hoc molecular phylogenetic methods and algorithms given that the available sequence data set of genes were currently very limited before the publication of the BEST program using the Bayesian method under the coalescence model [24]. However, we would like to point out that the species tree inferred from gene trees using the BEST program achieves high resolution, but is not always correct for all cases. The wrong species trees, such as those of ACLRS and MBCLRS, may be recovered from gene trees (Table 1). Therefore, the jackknife method for taxon sub-sampling is recommended to obtain further statistical confidence with jackknife support values. Additionally, this approach is newly published, it is not surprising that it has not been used widely in its early stage compared with the popular concatenation approach. Some caution should be kept. However, its use is strongly encouraged based on our study and other [27].

## Conclusions

This study provides strong evidence in favor of accepting Hypothesis 3, namely that the lungfish and coelacanth form a monophyletic sister group and that the sister group should be the phylogenetically closest living relatives of tetrapods. These conclusions are supported by high Bayesian posterior probabilities for the branch (a lungfish-coelacanth clade) and high taxon jackknife supports based on the genome-scale phylogenetic analysis of 43 genes using the latest program (BEST) [24,25,27] with the Bayesian method under the coalescence model and the jackknife method for taxon subsampling.

## Competing interests

The authors declare that they have no competing interests.

## Authors' contributions

YS and RG conceived and designed the experiments. YS analyzed the data. YS and RG wrote the paper. Both authors read and approved the final manuscript.

## Supplementary Material

Additional file 1 **Supplementary Table S1**. List of 43 gene names and their lengths (Number of amino acid positions encoded by the gene). Click here for file
