# Supplementary material for: 43 genes support the lungfish-coelacanth grouping related to the closest living relative of tetrapods with the Bayesian method under the coalescence model
Source: BMC Res Notes. 2011 Mar 7;4:49. doi: 10.1186/1756-0500-4-49 (PMC3069939; doi:10.1186/1756-0500-4-49)
Supplement: Additional file 1 — Supplementary Table S1. List of 43 gene names and their lengths (Number of amino acid positions encoded by the gene). [file 1756-0500-4-49-S1.DOC]

Supplementary Table S1. List of 43 gene names and their lengths (number of amino acid

positions encoded by the gene)

|  | Gene Name | Length |
| --- | --- | --- |
| 1 | ADH5 | 343 |
| 2 | ALAS1 | 318 |
| 3 | ALAS2 | 339 |
| 4 | ATP5A1 | 323 |
| 5 | CALR | 251 |
| 6 | CTSB | 199 |
| 7 | CCT8 | 213 |
| 8 | NR2F1 | 267 |
| 9 | PLP1 | 236 |
| 10 | EEF1G | 323 |
| 11 | FTH1 | 150 |
| 12 | GNB2L1 | 297 |
| 13 | HBA2 | 139 |
| 14 | HBB | 141 |
| 15 | NME2 | 116 |
| 16 | GPI | 268 |
| 17 | POMC | 76 |
| 18 | PRKCA | 378 |
| 19 | PRKCB1 | 271 |
| 20 | PTPN11 | 199 |
| 21 | RAG1 | 323 |
| 22 | RAG2 | 478 |
| 23 | RHO | 287 |
| 24 | RPL10A | 183 |
| 25 | RPL11 | 151 |
| 26 | RPL13 | 180 |
| 27 | RPL17 | 157 |
| 28 | RPL18 | 162 |
| 29 | RPL19 | 169 |
| 30 | RPL4 | 280 |
| 31 | RPL5 | 269 |
| 32 | RPL7 | 217 |
| 33 | RPL7A | 190 |
| 34 | RPL8 | 199 |
| 35 | RPLP0 | 252 |
| 36 | RPS3 | 211 |
| 37 | RPS4Y | 237 |
| 38 | RPS6 | 226 |
| 39 | RPS7 | 176 |
| 40 | RPS8 | 189 |
| 41 | RPS9 | 164 |
| 42 | TPI1 | 218 |
| 43 | TBP | 241 |

Notes: Gene names are those of human genes in the LocusLink of the National Center for Biotechnology Information (NCBI) database (http://www.ncbi.nlm.nih.gov/LocusLink/ ).
